# Fine-grained mathematical modeling for cost-effectiveness evaluation of public health policies for cervical cancer, with application to a Colombian case study

**DOI:** 10.1186/s12889-023-16022-x

**Published:** 2023-08-02

**Authors:** Daniela Angulo, Maria Fernanda Cortes, Ivan Mura, Raha Akhavan-Tabatabaei

**Affiliations:** 1 Department of Biostatistics, University of Michigan, Ann Arbor, Michigan United States; 2 Department of Industrial Engineering, Universidad de los Andes, Bogotá, Colombia; 3 Institute of Applied Physical Sciences and Engineering, and Global Health Research Center, Duke Kunshan University, Kunshan, China; 4 School of Management, Sabanci University, Istanbul, Turkey

**Keywords:** Cervical cancer, Public health policy, Cost-effectiveness analysis, Simulation model, Automation

## Abstract

**Background:**

Cervical cancer (CC) is globally ranked fourth in terms of incidence and mortality among women. Vaccination against Human Papillomavirus (HPV) and screening programs can significantly reduce CC mortality rates. Hence, executing cost-effective public health policies for prevention and surveillance is crucial. However, defining policies that make the best use of the available resources is not easy, as it requires predicting the long-term costs and results of interventions on a changing population. Since the simpler task of predicting the results of public health policies is difficult, devising those that make the best usage of available resources is an arduous challenge for decision-makers.

**Methods:**

This paper proposes a fine-grained epidemiological simulation model based on differential equations, to effectively predict the costs and effectiveness of CC public health policies that include vaccination and screening. The model represents population dynamics, HPV transmission within the population, likelihood of infection clearance, virus-induced appearance of precancerous lesions and eventually CC, as well as immunity gained with vaccination and early detection with screening.

**Results:**

We offer a compartmentalized modeling approach that separates population, epidemics, and intervention concerns. We instantiate models with actual data from a Colombian case study and analyze their results to show how our modeling approach can support CEA studies. Moreover, we implement models in an open-source software tool to simultaneously define and evaluate multiple policies. With the support of the tool, we analyze 54 policies within a 30-year time horizon and use as a comparator the CC policy that has been used until recently. We identify 8 dominant policies, the best one with an ICER of 6.3 million COP (Colombian Pesos) per averted DALY. We also validate the modeling approach against the available population and HPV epidemic data. The effects of uncertainty in the values of key parameters (discount rate, sensitivity of screening tests) is evaluated through one-way sensitivity analysis.

**Conclusions:**

Our modeling approach can provide valuable support for healthcare decision-makers. The implementation into an automated tool allows customizing the analysis with country-specific data, flexibly defining public health policies to be evaluated, and conducting disaggregate analyses of their cost and effectiveness.

## Background

Cervical cancer (CC) is the fourth most common cancer in women, with an estimated 604,000 new cases and 342,000 deaths around the world in 2020 [1]. This cancer type is almost always subsequent to an infection by the Human Papillomavirus (HPV). HPV infection is sexually transmitted, having a prevalence of up to 40% in young women [2]. Moreover, the estimated lifetime risk of contracting one or more genital HPV infections is about 80% for women [3].

Due to the magnitude of this public health issue, prevention and surveillance policies have been deployed worldwide. Primary prevention relies on vaccination against HPV. Although vaccines are generally very effective in preventing infection, they are not enough as the sole means against CC, and this is why surveillance (screening) plays a key role. Early treatment of precancerous lesions is highly effective, and it has been estimated that it can avert up to 80% of CC cases occurring in developing countries, which in turn account for 80% of the worldwide prevalence [4]. Cytology and HPV-DNA tests are two of the most common screening tests.

Many aspects affect the cost and utility of policies, for instance, the age range at which screening should start, the time interval between screenings and the type of tests to be applied. Similarly, there are options for vaccination that consider different target age ranges, and/or whether to vaccinate girls only or boys as well. The best settings are also likely to depend on country-specific aspects, such as demographics and socioeconomic factors. For this reason, fine-granularity predictive models, which can account for the details of interventions as well as the target population are of paramount importance when conducting Cost-Effectiveness Analysis (CEA) of CC interventions. Accurate and reliable models are even more crucial to CEA when there is a need to predict across long time horizons, as it is the case for CC.

Several types of predictive modeling approaches have been proposed to measure the cost-effectiveness of strategies against CC. In [5], the authors used a state-transition mathematical model to simulate a cohort of U.S women, and estimated a 93% reduction in lifetime risk by using liquid pap test for women up to 30 years old, and HPV and pap test for older women, with respect to a scenario without screening.

In [6], the authors developed a simulation model to study the natural history of CC using data from United Kingdom, The Netherlands, France and Italy. They evaluated the cost and health benefits of integrating HPV-DNA testing in CC screening programs. They found that strategies that incorporated HPV-DNA testing had lower cost, lower lifetime risk and higher life expectancy, compared to the current country strategies. Furthermore, in [7] the authors used Norway economic and epidemiological data for running a simulation model of HPV-induced CC. They compared the current Norway cytology scheme with strategies that included cytology for younger ages and HPV screenings for older ages. They concluded that the current scheme was less effective than the proposed interventions. In [8] the authors developed a differential-equation model that predicts the HPV vaccination impact on health and economics in the Sub-Saharan Africa. They found considerable health gains when pre-adolescent girls are vaccinated. For the Colombian population, in [9] a Markov model that represents the natural history of CC was developed in order to evaluate the effectiveness of different screening strategies. The authors defined an efficient frontier by comparing the costs and effectiveness of each strategy. Within the frontier, there were strategies that include vaccination and HPV-DNA test as the primary screening. An extended cost-effectiveness analysis of HPV vaccination was conducted in China by [10]. Using a Monte-Carlo simulation model, the authors estimated the distribution of deaths due to CC in scenarios that considered vaccination alongside screening. They found that if vaccination and screening were taken into account, cancer prevalence could decrease by 44%. In [11] a Markov Chain model was used to represent the natural history of the disease in Estonia, a country with low screening coverage. They evaluated the cost-effectiveness ratio of bivalent, quadrivalent and nonavalent vaccines in a school-based vaccination program. As a result, they found that vaccinating girls has an incremental cost-effectiveness ratio with any type of vaccination scheme, compared to current programs. Moreover, bivalent vaccines had the lowest incremental cost, and nonavalent vaccines the highest QALYs gained.

The results of the studies reported above support the need of capturing country-specific aspects into models that can predict the cost-effectiveness of public health policies against CC. In general, these studies evaluate the cost-effectiveness of selected policies for a given context, such as a country or a set of countries. Thus, repeating the study for different policies or countries would require a substantial amount of data preparation and additional modeling.

The objective of this work is to define a general and rigorous modeling methodology that can predict relevant metrics to be used in CEA studies of CC interventions for the population of a country. The modeling supports the integration of country-specific data, the analysis of a large class of vaccination and screening interventions, the estimation of incidence, prevalence and death rates, and the generation of cost-effectiveness relevant metrics. Moreover, we also offer a software application that can automate most of the modeling and outcome metric evaluation steps.

Our approach is based on mathematical models that comprehend the health states dynamics, and use compartmentalized epidemiological models, rendered in form of ordinary differential equations. The numerical integration of differential equations can reproduce the dynamics of the population, the transmission of HPV and the possible progression to CC stages, under the effects of defined vaccination and screening policies. To provide a flexible solution for public health decision-makers, our modeling was automated into an open software tool, developed with R^®^ [12]. The software tool is endowed with a Shiny^©^ [13] graphical user interface, which almost completely hides the modeling complexity from the final user.

The paper is organized as follows. In the Methods section we present the modeling methodology used for the CEA of policies against CC, the data required, and the software tool developed for automating the implementation. In the same section, we present an application of the modeling approach to the study of CC interventions on the Colombian population. The Results section includes a partial validation of the proposed modeling methodology and some elements of evaluation of cost and effectiveness for 54 policies that could be of interest for the Colombian use case. The Discussion section conducts a critical review of our proposal, its findings, and its limitations. Finally, the Conclusions section presents our main remarks and perspectives for future research work.

## Methods

Since this work aims at modeling the disease spread (HPV infection and progression to CC) at a population level, we base our modeling on classical, deterministic continuous-time epidemics modeling [14]. This approach partitions the whole population into a discrete number of subsets, called *compartments*, each containing individuals who can be considered homogeneous with respect to the variables that affect the dynamics of the disease. The change in the number of individuals within compartments is expressed in terms of a set of ordinary differential equations. Given the initial state provided at time $t=0$, the numerical integration of the differential equations provides a prediction for the population state over time.

We choose continuous deterministic modeling because the large number of entities (the whole population of a country) is ensuring that a continuous approximation is adequate for the number of individuals in the system. Moreover, this type of model is very efficient and allows obtaining predictions over long time horizons (30 to 50 years) in a limited computing time.

Models that can accurately reflect the dynamics of HPV and CC need to consider demographic, socio-economic, and epidemiological aspects. To handle modeling complexity, we separately represent three distinct aspects or *modeling layers*:

- The *population layer*, where we model the dynamics of population without considering the disease. The processes affecting the number of individuals in compartments will be births, deaths and aging.
- The *disease layer*, which models the transmission and clearance of HPV infection within the population, and the possible transitions to pre-cancerous and cancerous stages.
- The *intervention layer*, in which we model the impact of prevention and/or surveillance actions that counter HPV epidemics, and detect and control the progression of CC lesions.

**Fig. 1:**
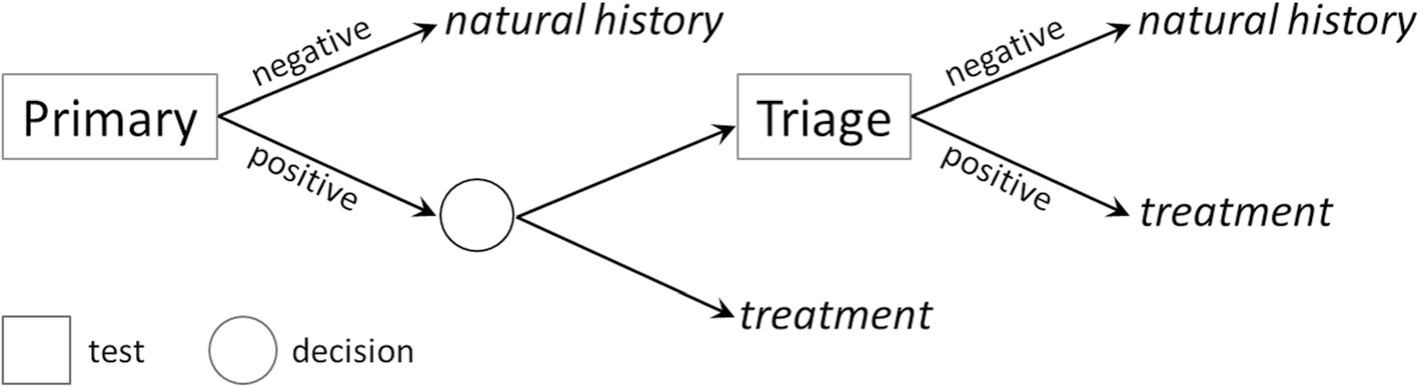
Triage screening policies scheme

Each model layer is built on top of the previous one, by refining the compartmentalization with a finer subdivision of the population. The population layer can be parameterized using official census data on the number of births and deaths in the population, and validated using other population projections, for instance, those provided by the United Nations (UN). For the second layer, country-specific data on HPV incidence, CC incidence and CC death rates is required. Validation can be conducted by comparing model predictions against health records for disease prevalence in the population. For the last layer, the parameterization requires defining details of the vaccination and/or screening policies to be evaluated, as well as their effectiveness, e.g. immunity acquired, quality of the screening tests, etc. For validation, the results of previous cost-effectiveness literature studies can be used. The third modeling layer specifies the prevention and surveillance interventions that decision-makers are interested in evaluating. Among the possible prevention actions, we focus on vaccination. We assume vaccination campaigns are defined according to a *pulse* vaccination strategy, by which a fraction of the susceptible population is vaccinated in a single pulse applied every certain periods [15]. Our model considers that vaccinated individuals can be from any gender and age, according to the inputs provided by the decision-makers. As for surveillance, we consider the family of screening policies that consist of primary and triage tests, as depicted in Fig. 1.

In this type of screening, a positive result obtained by a primary test will have to be confirmed by another (*triage*) test. This double check will be of course not necessary in those circumstances when a certain diagnosis can be done with the primary test alone.

Policies will be evaluated in terms of cost and effectiveness. In this work, the cost of a policy is the sum of the direct costs for vaccination and screening, considering a health system perspective. As for the effectiveness, we adhere to WHO recommendations for cost-effectiveness analysis in developing countries [16] by using Disability Adjusted Life Years (DALY). DALYs combine mortality and morbidity into a single metric: they are the sum of the potential years of life lost due to premature death, and the equivalent years of healthy life lost due to unhealthy life conditions.

In the next subsections we provide the details of the compartmentalized modeling approach we will be using. We proceed bottom up to describe the three modeling levels introduced in the previous section, and finally we model cost-effectiveness. We only provide the modeling rationale in the main body of the paper, moving to a final section several mathematical details to keep the reading lean and easy to follow.

### Population layer model

Even slow changes in the composition of a population can become relevant over long time horizons. Therefore, we envisaged the need to build epidemics models on top of reliable predictive models of population dynamics.

For the population layer model, we divide the population into genders (females and males), and age ranges. The number of age divisions is the modeler’s decision. We denote by $A =\{0,1,\ldots ,K\}$ the set of age ranges and we assume each age range in *A*, measured in years, will span the same temporal width, except for the last range *K* which will account for all individuals in the tail of the age distribution. Let $F_a(t)$ and $M_a(t)$ denote the number of women and men in age range $a \in A$, at time $t, t > 0$. We will consider $F_a(t)$ and $M_a(t), a \in A$, to be continuous variables of our model. This is clearly an approximation, which disregards the true discrete nature of a counting variable, but it is however commonly accepted due to the simplicity it entails in studying the population dynamics. The changes that can affect these continuous variables include births, aging and deaths, which will be modeled as differential equations.

Births will affect the number of individuals in the first age range, i.e. $F_0(t)$ and $M_0(t)$. To characterize the birth process we introduce the time-dependent fertility rate of women at age range *a*, denoted by $\pi _a(t)$, which provides the average number of children a woman in age range $a \in A$ gives birth per year, at time *t*. Also, we denote by $p_f$ the probability that a newborn is female, which we assume has no dependence on age range nor on time.

The term $\sum _{a \in A} F_a (t)\pi _a (t)$, which we shall denote as *N*(*t*) hereafter, is the rate of births (individuals per year) in the population at time *t*. Population aging is modeled as a constant rate of change, with individuals moving from one age compartment to the next, for all age ranges, except the last one. The rate of aging, denoted by $\theta$ is the inverse of the age range width. To characterize death dynamics for all causes, we introduce the time-dependent death rate of individuals in age range $a \in A$ at time *t*, which we shall denote by $\mu ^F_a(t)$ and $\mu ^M_a(t)$ for women and men, respectively. The differential equations, including births, aging and deaths are presented below. For the first age range:

1 $$\begin{aligned} \frac{dF_0(t)}{dt}= & {} N(t)p_f - \theta F_0(t) - \mu ^F_0(t) F_0(t) , \nonumber \\ \frac{dM_0(t)}{dt}= & {} N(t) (1-p_f) - \theta M_0(t)- \mu ^M_0(t) M_0(t) \end{aligned}$$

For all age ranges *a*, with $0< a < K$:

2 $$\begin{aligned} \frac{dF_a(t)}{dt} = \theta F_{a-1}(t) - \theta F_a(t) - \mu ^F_a(t) F_a(t), \quad \nonumber \\ \frac{dM_a(t)}{dt} = \theta M_{a-1}(t) - \theta M_a(t)- \mu ^M_a(t) M_a(t) \end{aligned}$$

Finally, for the last age compartment we have the following two differential equations:

3 $$\begin{aligned} \frac{dF_K(t)}{dt}= \theta F_{K-1}(t) - \mu ^F_K(t) F_K(t), \quad \frac{dM_K(t)}{dt}= \theta M_{K-1}(t) - \mu ^M_K(t) M_K(t) \end{aligned}$$

The mathematical formulation of the differential equations can be streamlined by introducing auxiliary indicator functions, as explained in Mathematical modeling details section at the end of the paper. We do not consider incoming or outgoing immigration flows in this model. However, including them will not change the fundamental structure of the population model. The sets, variables and parameters used in the model are summarized in Table 1.

**Table 1:** Sets, variables and parameters used in the population model

**Sets**	**Description**
A	Age ranges
**Variables**	**Description**
$F_a(t)$	Number of women of age range $a \in A$ at time *t*
$M_a(t)$	Number of men of age range $a \in A$ at time *t*
**Parameters**	**Description**
$\pi _a(t)$	Fertility rate of women in age range $a \in A$ at time *t*
$p_f$	Probability that a newborn is a woman
$\mu ^F_a(t)$	Death rate of women in age range $a \in A$ at time *t*
$\mu ^M_a(t)$	Death rate of men in age range $a \in A$ at time *t*
$\theta$	Population aging rate

### Disease layer modeling

We refine the population dynamics by partitioning each compartment into smaller ones, so that individuals in each compartment are homogeneous not only for gender and age, but also for health state. Relevant health states in our modeling include those that characterize HPV transmission dynamics (e.g. healthy, infected), and the various disease states determined by the consequences of the infection (e.g. different grades of CIN or stages of CC). The set of possible health states for males, denoted by *HM*, is a subset of the females health states set, denoted by *HF*.

In this layer of modeling, we use $F_a^h(t)$ to denote the number of women of age $a \in A$ and health state $h \in {HF}$ at time *t*; and $M_a^h(t)$ to denote the number of men of age $a \in A$ and health state $h \in {HM}$ at time *t*. Individuals in the population will change their health state compartment according to the natural evolution of the HPV infection and the natural history of CC evolution. The rate at which individuals get infected with HPV depends on their gender and age, which determine their approximate number of sexual partners per year and the likelihood that their sexual partners are HPV infected. This latter factor can be estimated as the ratio of infected potential partners and the total number of potential partners, which in our model evolve as functions of time. We assume that within each of the compartments defined in the population layer model, all individuals are homogeneous with respect to their sexual behavior, so that the rate of HPV infection at time *t* is the same for all the individuals in the compartment.

To capture the dynamics of health state change, we introduce the matrix of transition rates for women $\mathbb{P}\mathbb{F}_a(t)$. This matrix has dimensions $|{HF}\times {HF}|$, and its entry $h_1,h_2$ (denoted as $\mathbb{P}\mathbb{F}_a^{h_1,h_2}(t)$) is the rate with which a woman in age range $a \in A$ and health state $h_1 \in {HF}$, changes her health state to $h_2 \in {HF}$ at time *t*. For men, we define the analogous matrices of functions $\mathbb{P}\mathbb{M}_a(t)$, $a \in A$, each having dimensions $| {HM}\times {HM}|$. With such rate functions, we can describe the evolution of the number of individuals in their health states (no births, no change of age range, no deaths), as follows:

4 $$\begin{aligned} \frac{dF_a^{h_1}(t)}{dt} =\sum \limits _{h_2 \in {HF}} F_a^{h_2}(t)\mathbb{P}\mathbb{F}_a^{h_2,h_1}(t) - \sum \limits _{h_2 \in {HF}} F_a^{h_1}(t)\mathbb{P}\mathbb{F}_a^{h_1,h_2}(t) ,\ a \in A, \, h_1 \in {HF} \end{aligned}$$

5 $$\begin{aligned} \frac{dM_a^{h_1}(t)}{dt} =\sum \limits _{h_2 \in {HM}} M_a^{h_2}(t)\mathbb{P}\mathbb{M}_a^{h_2,h_1}(t) - \sum \limits _{h_2 \in {HM}} M_a^{h_1}(t)\mathbb{P}\mathbb{M}_a^{h_1,h_2}(t) , a \in A, \, h_1 \in {HM} \end{aligned}$$

In each of the equations above, the terms in the positive summation account for the individuals who change their health state to become $h_1$, and the terms in the negative summations for those who change their health state to become a health state other than $h_1$.

Since perinatal transmission of HPV is not uncommon [17], it is necessary to characterize the health state at birth. We thus introduce vector $\mathbb{B}\mathbb{F}$ for females and $\mathbb{B}\mathbb{M}$ for males, whose entries provide the probability distribution for the initial health state. The interested reader can find the final form of the differential equations in the Mathematical modeling details section. Sets, variables and parameters introduced in this modeling layer are summarized in Table 2.

**Table 2:** Sets, variables and parameters used in the disease model

**Sets**	**Description**
HF	Health states of females
HM	Health states of males
**Variables**	**Description**
$F_a^h(t)$	Number of women of age $a \in A$ in health state $h \in {HF}$ at time *t*.
$M_a^h(t)$	Number of men of age range $a \in A$ in health state $h \in HM$ at time *t*.
**Parameters**	**Description**
$\mathbb{P}\mathbb{F}_a(t)$	Matrix of transition rates between health states for women of age $a \in A$ at time *t*.
$\mathbb{P}\mathbb{M}_a(t)$	Matrix of transition rates between health states for men of age $a \in A$ at time *t*.
$\mathbb{B}\mathbb{F}$	Vector of health state probability distribution at birth for women.
$\mathbb{B}\mathbb{M}$	Vector of health state probability distribution at birth for men.
$\mu ^F_{a,h}(t)$	Death rate for women of age $a \in A$ and health state $h \in {HF}$, at time *t*.
$\mu ^M_{a,h}(t)$	Death rate for men of age $a \in A$ and health state $h \in {HM}$, at time *t*.

### Intervention layer modeling

Finally, we define the intervention layer model, which considers vaccination and screening as possible prevention and surveillance policies. The effect of interventions is modeled through the change they exert on the rates of health state transition functions $\mathbb{P}\mathbb{F}_a(t)$ and $\mathbb{P}\mathbb{M}_a(t)$.

#### Modeling vaccinations

To model a pulse vaccination intervention, the percentage of individuals to be vaccinated, their ages and the duration of the intervention (in years) must be defined. During each year, several ages can be the target of the vaccination campaign.

We assume that vaccination is effective only if applied to individuals that have not yet been exposed to HPV. We denote by $HF _V$ the subset of health states $HF$ of women for which the vaccination would be effective, and by $HM _V$ the analogous subset of health states for men.

We assume that when individuals whose health state is in $HF _V$ (for women) or in $HM _V$ (for men) are vaccinated, permanent immunity against HPV is acquired. Therefore, in this modeling layer we add a new health state $V$ to keep track of the immunized sub-population. Also, we define new parameters ⓕ$_a^{y(t)}$ and ⓜ$_a^{y(t)}$ to respectively represent the percentage of women and men, of age $a \in A$ to be vaccinated in year *y*(*t*) of the intervention. The differential equations for this modeling layer are only reported in the Mathematical modeling details section. Table 3 reports the sets, variables and parameters introduced for modeling the vaccination interventions.

**Table 3:** Sets, variables and parameters introduced for modeling vaccinations

**Sets**	**Description**
$HF_V$	Subset of health states *HF* for which the vaccination would be effective
$HM_V$	Subset of health states *HM* for which the vaccination would be effective
**Variables**	**Description**
$F_a^V(t)$	Number of women of age $a \in A$ who have been immunized by time *t*
$M_a^V(t)$	Number of men of age range $a \in A$ who have been immunized by time *t*
**Parameters**	**Description**
ⓕ$_a^{y(t)}$	Target percentage of women of age $a \in A$ to be vaccinated in year *y*(*t*)
ⓜ$_a^{y(t)}$	Target percentage of men of age $a \in A$ to be vaccinated in year *y*(*t*)

#### Modeling screening

The definition of a triage screening policy requires selecting the primary and the secondary tests, the ages at which the screening is to be performed, and its frequency. To model screening policies, we consider a possible set $\Phi$ of tests, and each test $\phi _i \in \Phi$ will be characterized by its sensitivity $\overline{\beta }_i$ and specificity $\overline{\alpha }_i$.

**Fig. 2:**
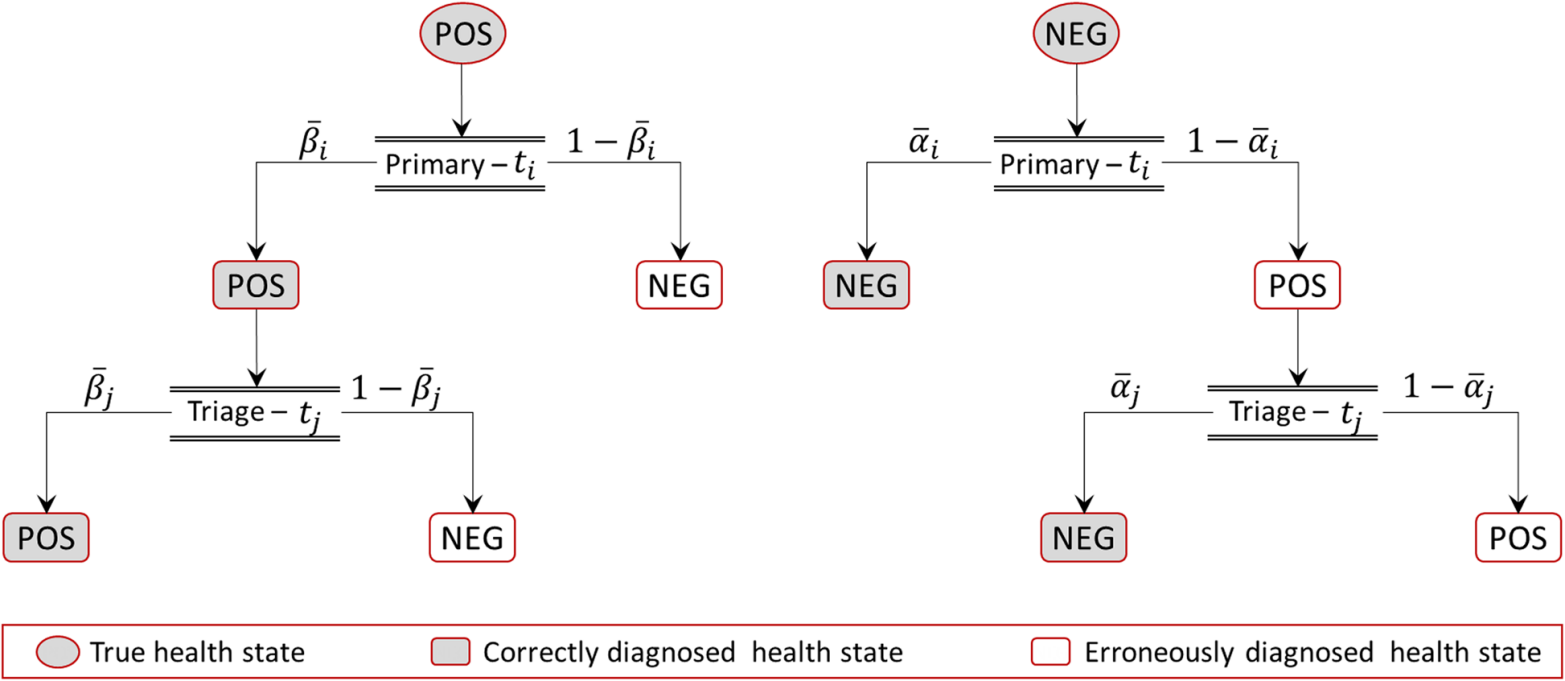
Diagnosis in triage screening schemes

In a triage screening scheme, the diagnosis follows the process shown in Fig. 2. The likelihood of a correct diagnosis depends on the sensitivity and specificity of the two tests used in the screening. A triage screening strategy (using test $\phi _i$ as primary and $\phi _j$ as secondary) will provide a correct classification for true positive individuals with probability $c_{i,j}^{+}= \overline{\beta }_i\overline{\beta }_j$. For true negative individuals, the probability of correct classification is $c_{i,j}^{-}= 1-(1-\overline{\alpha }_i)(1-\overline{\alpha }_j)$.

We introduce $A_S \subset A$, the subset of the ages to apply screening to, and $f_a$, the screening frequency (in screenings/year) at age $a \in A$. Moreover, adherence of women to screening policies is an important aspect, as historical data shows that only a fraction of the target thoroughly follows it. The underlying reasons are multi-faceted and country specific, see for instance [18, 19]. For the sake of our modeling we will introduce the age dependent parameter $d_a$, whose value provides the percentage of women of age $a \in A_S$ who adhere to the screening program.

When a CIN or CC condition is diagnosed, an intervention may be necessary. We consider two distinct types of interventions: local treatment (excision and ablation) of CIN lesions, and hysterectomy for CC stages. For considering this, we define ${HF}_{L}$ and ${HF}_{H}$, two disjoint subsets of *HF*: the first one considers the health states that, when diagnosed, will lead to local treatment; and the latter those that will lead to hysterectomy. A new health state $\ddot{h}$ is introduced to keep track of women who underwent hysterectomy. The differential equations are shown in the Mathematical modeling details section. Table 4 provides a summary of the set, variables and parameters introduced for modeling screening.

**Table 4:** Set, variables and parameters used to model screening interventions

**Sets**	**Description**
$\Phi$	Set of available tests
$A_S$	Subset of the ages ranges *A* where screening is applied to
$HF_L$	Subset of health states *HF* for which when diagnosed will lead the individual to local treatment
$HF_H$	Subset of health states *HF* for which when diagnosed will lead the individual to hysterectomy
**Variables**	**Description**
$F_a^{\ddot{h}(t)}$	Number of women of age $a \in A$ who underwent an hysterectomy, at time *t*.
**Parameters**	**Description**
$\overline{\beta }_i$	Sensitivity of test $\phi _i$.
$\overline{\alpha }_i$	Specificity of test $\phi _i$.
$c_{i,j}^{+}$	Overall sensitivity, when using $\phi _i$ as primary and $\phi _j$ as secondary.
$c_{i,j}^{-}$	Overall specificity, when using $\phi _i$ as primary and $\phi _j$ as secondary.
$f_a$	Frequency of screening for women of age $a \in A$ defined by the screening policy.
$d_a$	Percentage of women of age $a \in A$ who will adhere to the screening policy.

### Cost-effectiveness modeling

Cost-effectiveness analysis (CEA hereafter) provides a suitable framework for quantitatively estimating and comparing the total impact, in terms of both health and economic consequences, of different policies. For measuring cost-effectiveness we use a *C*/*E* ratio [20] that measures the merits of a new intervention with respect to *baseline scenarios* or *comparators*. The comparator is typically representative of the currently applied public health policy. The two elements of the *C*/*E* metric are defined as follows:

- *C* is the incremental cost of the resources consumed with the adoption of the new interventions, with respect to the comparator.
- *E* is health improvement generated with the adoption of the new interventions, with respect to the comparator.

The health outcome can be quantified in a variety of ways. In the following, we consider DALYs [21]. When effectiveness is measured through DALYs, the ratio *C*/*E* is usually referred as *cost per the DALY averted*, because it estimates the additional expenditure necessary to save one DALY. Distinct interventions are compared through their *C*/*E* ratios, and in the analysis interventions with the smallest ratios are the most promising ones. Though, a disaggregated analysis that evaluates the trade-offs between cost and effectiveness is also conducted, to identify public health policies that may offer additional advantages.

#### Estimating costs

We assume that the total cost of a public CC prevention and surveillance policy corresponds to the sum of the direct costs bore by the health-care payer for vaccination and screening over [0, *T*], the defined time horizon for the analysis.

The total vaccination cost ($C_V$) of a policy is calculated by multiplying the unitary cost of vaccination doses (reported to present costs) by the number of vaccinations administered over [0, *T*]:6where *v*(*t*) is the time-dependent function that models the net-present cost of the vaccination doses, and the expression in the summation corresponds to the instantaneous rate of vaccination at time $t \in [0,T]$.

The total screening cost ($C_{S}$) is calculated as the number of tests performed in a given time frame [0, *T*], multiplied by the cost per test. We separately model the costs of primary and secondary screening tests, which we denote by $C_{PS}$ and $C_{SS}$, respectively.

7 $$\begin{aligned} C_{PS} = \int _0^T \sum \limits _{a \in {A}_{S}} \sum \limits _{h \in {HF}} \left[ F^{h}_a(t)f_a d_a \right] s_i(t) dt \end{aligned}$$

Variable $s_i(t)$ is the direct cost of primary screening test $\phi _i$ at time *t*, and the summation corresponds to the instantaneous rate of primary screening.

Similarly, the triage screening costs $C_{SS}$ are calculated as the number of triage tests performed in the time frame [0, *T*], multiplied by the unitary cost $s_{j}(t)$ of the applied test $\phi _{j}$ at time *t*. As a triage test is done after a positive result of a primary test $\phi _{i}$ without regard to the patient’s true state, we need to sum the costs incurred for the right diagnosis of true positive cases, as well as those of the misdiagnosis of negative individuals, i.e. the primary false positives:

8 $$\begin{aligned} C_{SS} = \int _0^T \sum \limits _{a \in {A}_{S}} \left[ \sum \limits _{h_1 \in {HF}_T} F^{h_1}_a(t)f_a d_a \overline{\beta }_i + \sum \limits _{h_2 \notin {HF}_T} F^{h_2}_a(t)f_a d_a (1- \overline{\alpha }_i)\right] s_j(t) dt, \end{aligned}$$

where ${HF}_T = {HF}_L \cup {HF}_H$ is the set of all health states of true positive women.

#### Estimating effectiveness

To estimate the effectiveness, we separately evaluate the DALYs accrued as a consequence of premature deaths due to CC (Years Lost Life - YLL) and those for the time spent in disease states (Years Lived with Disability - YLD).

The YLL component of the total DALYs is computed by summing up the total contribution of deaths that occur at each age $a\in A$, across the time horizon [0, *T*]. If a woman in age range *a* dies of CC at time *t*, the contribution to the YLL component of DALYs will be $max\{0,L-\overline{a}\}$, where *L* is the expected lifetime of women and $\overline{a}$ is the center of the age interval *a*. The exact computation of the YLL would require considering the precise death age within the range *a*. Since we represent the age as a range, the computation of the DALYs will be approximate.

For each age range *a* such that $\overline{a} \le L$ we consider an additional health state $\ddot{d}$ for death. Let $F^{\ddot{d}}_a(t)$ be the continuous variable that accumulates the number of individuals who have died of CC at age *a* by time $t \in [0,T]$. Then, the values of $F^{\ddot{d}}_a(T)$ will give the total number of women who have died of CC across the whole time horizon [0, *T*], at each age $a\in A$, and the total number of YLLs for the whole population can be estimated as follows:

9 $$\begin{aligned} YLL = \sum \limits _{a\in A, \overline{a} \le L} F^{\ddot{d}}_a(T)\left( L-\overline{a}\right) \end{aligned}$$

Notice that approximating the true time-dependent expected life-time by a constant *L* can introduce a significant approximation over large time horizons. The expression in Eq. 9 can be easily modified to account for a piece-wise constant approximation of any *L*(*t*) function.

We consider the following health states to be YLD contributors:

- $HF_L \cup HF_H$, i.e. states that would require treatment;
- *cc*, the cervical cancer state;
- $\ddot{h}$, the state entered after hysterectomy.

Each state above is mapped to a specific weighing factor, which accounts for the severity of impairment in the ability of the individual in conducting a normal life. Let us denote by $W_h$ the weight factor of a health state *h*.

To compute the YLDs, we introduce dedicated compartments and variables in the model. For each YLD contributor state *h*, a continuous variable $YLD_h(t)$ is added to the model, whose value corresponds to the accumulated YLD for all the individuals that spent a time interval in health state *h*. The differential equation that describes the evolution of variable $YLD_h(t)$ is:

10 $$\begin{aligned} \frac{d}{dt}YLD_h(t) = \sum \limits _{a\in A} W_h F^{h}_a(t). \end{aligned}$$

The value $YLD_h(T)$ at the end of the time horizon gives the total accumulated YLDs for each state $h \in HF$ and for state *cc*.

The contribution of state $\ddot{h}$ is to be computed with a different approach. Since a woman undergoing hysterectomy will suffer a permanent impairment of her reproductive health, YLDs will be accrued from the moment an individual enters the state $\ddot{h}$ until the end of the woman’s fertile period (as estimated from the population). The age *a* at which women undergo hysterectomy is tracked by a dedicated variable $F^{\ddot{h}}_a(t)$, and the estimation of this YLD component is then carried out with computations similar to the one described in Eq. 9 for the YLL. Table 5 summarizes the variables and parameters introduced for cost and effectiveness modeling.

**Table 5:** Variables and parameters introduced for modeling cost and effectiveness

**Variables**	**Description**
$C_V$	Total cost of vaccinations over the time horizon [0, *T*]
$C_{PS}$	Total cost of primary screening tests over the time horizon [0, *T*]
$C_{SS}$	Total cost of secondary screening tests over the time horizon [0, *T*]
$F_a^{\ddot{d}}(t)$	Number of Women who died of CC in age range $a \in A$, up to time *t*
$YLD_h(t)$	YLDs accumulated up to time *t* for individuals who lived in health state $h \in HF$
**Parameters**	**Description**
*L*	Women lifetime expectancy in the population
$W_h$	Weight factor for health state $h \in HF$, used for YLD estimation
$c_{i,j}^{+}$	Overall sensitivity, when using $\phi _i$ as primary and $\phi _j$ as secondary
$c_{i,j}^{-}$	Overall specificity, when using $\phi _i$ as primary and $\phi _j$ as secondary
$f_a$	Frequency of screening for women of age $a \in A$ defined by the screening policy
$d_a$	Percentage of women of age $a \in A$ who will adhere to the screening policy
*v*(*t*)	Direct cost of vaccination per person at time *t*
$s_{i(t)}$	Direct cost of test $\phi _i$ at time *t*

## Results

We present here results from the application of the proposed modeling approach to the analysis of a hypothetical set of public health policies that could be deployed against CC in Colombia. While carrying out a complete and rigorous CEA is out of our scope, we want to demonstrate that our modeling approach can provide many of the quantitative predictions required by decision-makers for a detailed CEA study.

The case of Colombia is an interesting one because eventhough significant progress has been made in reducing the mortality for CC [22], from 14 deaths per 100,000 women in 1987 to 7.08 deaths per 100,000 in 2013 [23, 24], a very heavy burden of the disease was estimated for 2016 [25]. Also, the country has recently introduced a new public health policy against CC, which includes combined vaccination and screening.

Vaccination against HPV was introduced in Colombia in 2012. Girls aged between 9 to 17 years [26] are the target of the immunization campaigns. Screening against CC has also been in place in Colombia since 1990, following the 1-1-3 scheme. This scheme requires annual collection and analysis of a pap-smear specimen. After two consecutive years with negative results, the frequency of the cytology decreases to once per 3 years. However, with the introduction of the HPV-DNA test, efforts have been made to introduce the HPV DNA test as a primary screening test since 2017 [27, 28]. Considering that the HPV-DNA test has a higher sensitivity [29], it may provide better health benefits; however, it is also more expensive than cytology.

The cost-effectiveness of HPV DNA test versus cytology for CC screening has been studied in Colombia [30], but the the best of our knowledge, no study has yet considered the combined effects of policies that include both prevention and surveillance interventions. This motivates us to analyze and evaluate the costs and health benefits of different prevention and surveillance policies against CC in the country, taking as a comparator the policy that has been used in the country until recent years. This analysis also serves the purpose of showing the open-source application we developed for its automation.

We first configure the study, detailing the choice made for compartmentalization. Then, we discuss the model initialization and parameterization, briefly mentioning the used data sources. To assess the study quality, we applied the Consolidated Health Economic Evaluation Reporting Standards (CHEERS) checklist [31]. The assessment included the study perspective, comparator, outcomes, time horizon, cost discounting, reporting of the target population, sensitivity analysis, among others.

### Configuring the study

**Fig. 3:**
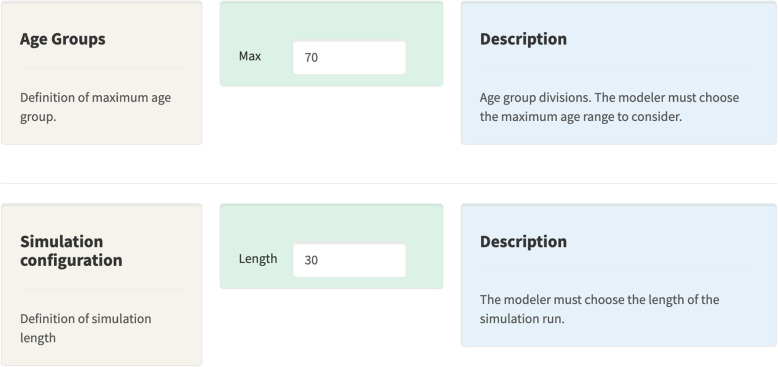
Age ranges configuration tab

We apply our modeling approach by partitioning the population by gender, and each gender further into 70 disjoint age ranges: $A=\{0,1,...,70\text { or older}\}$. Figure 3 shows a screenshot of the automated tool, through which a user can specify the maximum age, and simulation length. The range corresponding to the maximum age includes all individuals that are at least 70.

As for the time horizon, we choose a 30-year period, which starts in 2020. Such a wide time window is needed to ensure that the mid- and long-term benefits of vaccination campaigns are observable in the population.

### Model initialization and parameterization

To initialize the state of the model, we assign the initial population in each age range and gender using data from the National Department of Statistics (DANE) population projections for 2020 [32]. For females, we considered health states that included the precancerous stages CIN1, CIN2 and CIN3. We do not detail CC stages, as we are not considering treatments apart from hysterectomy. Other choices are of course possible and our modeling approach can easily accommodate them. For the prevalence of the HPV infection in men and women, we obtain data from [33]. For the prevalence of the CIN stages and CC among women, we use the studies done by [34–37]. We assume that all newborns are in the healthy state.

To estimate the number of immunized individuals at time $t=0$, we use data provided by the Colombian Ministry for Health, on approximately 2.13 million girls aged between 9 and 19 who were subject to HPV vaccination between 2012 and 2019. The national campaign was only directed and subsided to girls. Thus we do not consider immunized boys in the initial state of the model. We calculated the percentage of hysterectomized women of each age range with data from a Colombian survey ([38]). We also used information from the German Health Interview and Examination Survey for Adults [39]. The number of individuals in all other compartments, including those for deaths and DALYs estimation, will be initialized in zero.

We now describe the parameters of the equations following our layering scheme, in a bottom-up fashion. In the population layer, the age-dependent birth and mortality rates were estimated based on the population statistics published annually by DANE [32]. With a double exponential smoothing [40] we extrapolated time-dependent functions for the fertility rates and for death rates across the analyzed time-horizon. The probability that a newborn is female was estimated from the Colombian birth data available in [41].

For the disease layer, we estimated the HPV infection rates from the age-dependent number of sexual partners for Colombian men and women during a year [38], the likelihood of contracting the HPV infection during one sexual intercourse [42], and the proportion of infected individuals over the total population. The remaining natural history health state transition rates were estimated from five years of CC screening medical records from a Colombian private health service provider [43, 44]. Finally, we obtained the CC mortality rates from a population-based cancer research study conducted in Colombia [45].

The parameters of the intervention layer define the policies for the CEA. The comparator considers pulse vaccination for five years since $t=0$, with 6% of girls aged between 9 and 19 years vaccinated per year (around 40,000 girls vaccinated per year, as the current vaccination campaign). Vaccination is combined with a 1-1-3 screening scheme used throughout the whole time horizon of the analysis, where cytology is the primary and HPV-DNA the secondary test.

We consider a set of possible policies that combine vaccination and screening. For vaccination, in all considered cases the target population is composed by individuals aged between 9 and 19. We analyze 11 pulse vaccination strategies reported in Table 6, which are characterized by the target gender, the percentage of the target to vaccinate, and the duration of the intervention. The dummy intervention *Vac0* stands for no vaccination, and *Vac1* is the current vaccination intervention.

**Table 6:** Vaccination interventions

ID	% target women	% target men	Time window
Vac0	0%	0%	year 1 - year 30
Vac1	6%	0%	year 1 - year 5
Vac2	10%	10%	year 1 - year 5
Vac3	0%	10%	year 1 - year 5
Vac4	10%	0%	year 1 - year 5
Vac5	0%	20%	year 1 - year 5
Vac6	20%	0%	year 1 - year 5
Vac7	20%	20%	year 1 - year 5
Vac8	20%	20%	year 1 - year 10
Vac9	20%	20%	year 1 - year 15
Vac10	20%	20%	year 1 - year 20

**Fig. 4:**
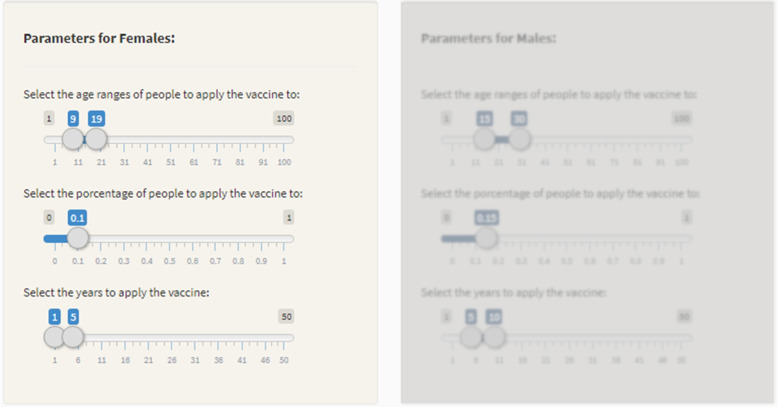
Vaccines intervention definition tab: defining Vac4

Figure 4 shows the software tab for defining vaccination interventions. The user can select the target gender, ages, percentages, and the duration of the intervention. In Fig. 4, intervention *Vac4* is defined, which only considers vaccination for girls.

Table 7 reports the screening interventions considered. All of them use cytology and HPV-DNA as primary or secondary tests, and they are applied throughout the whole time horizon. We considered the following frequency schemes:

1-1-3 : test now, second test after one year, third test after another year, and subsequent tests every 3 years;
1-5 : test now, second test after one year, and subsequent tests every 5 years;
3-5 : test now, second test after 3 years, and subsequent tests every 5 years;
3 : a fixed frequency scheme, testing every 3 years.

Intervention *Sc1* denotes the comparator screening policy.

**Table 7:** Screening interventions

ID	Primary test	Triage test	Screening frequency scheme
Sc1	Cytology	HPV-DNA	1-1-3
Sc2	HPV-DNA	HPV-DNA	1-1-3
Sc3	HPV-DNA	HPV-DNA	3-5
Sc4	Cytology	HPV-DNA	3-5
Sc5	Cytology	HPV-DNA	3
Sc6	HPV-DNA	HPV-DNA	3
Sc7	HPV-DNA	HPV-DNA	1-5

**Fig. 5:**
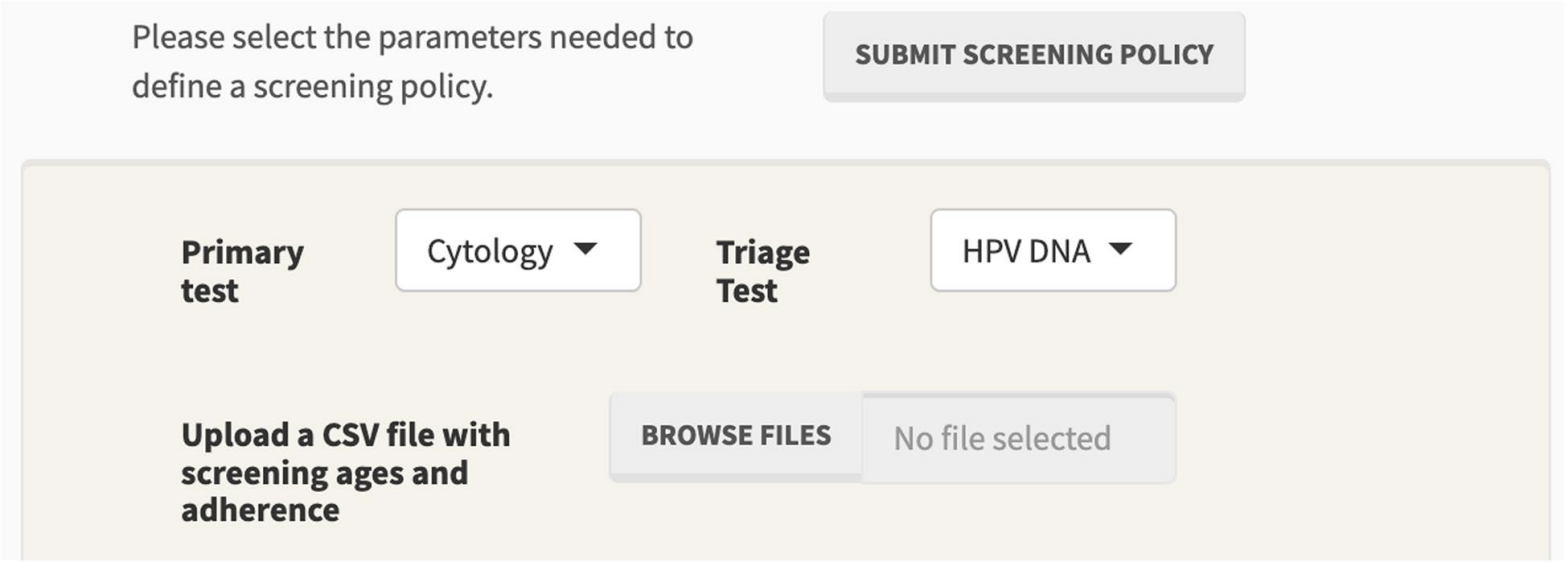
Screening intervention definition tab: defining Sc1

Figure 5 shows the software tab for defining screening interventions. The user is allowed to choose which tests to use as primary and triage, and to upload a file with the ages in which screening is applied, along with the estimated adherence.

The combination of vaccination and screening interventions define 54 possible policies, which are shown in Table 8. Notice that policy P1 corresponds to the comparator for the purpose of our analyses. The cost per averted DALY of policies P2-P54 will be compared with respect to those of policy P1.

**Table 8:** Public health policies compared to P1

ID	Vacc	Scr	ID	Vacc	Scr	ID	Vacc	Scr
P1	Vac1	Sc1	P19	Vac0	Sc4	P37	Vac3	Sc6
P2	Vac2	Sc1	P20	Vac2	Sc4	P38	Vac4	Sc6
P3	Vac3	Sc1	P21	Vac3	Sc4	P39	Vac5	Sc6
P4	Vac4	Sc1	P22	Vac4	Sc4	P40	Vac6	Sc6
P5	Vac5	Sc1	P23	Vac5	Sc4	P41	Vac7	Sc6
P6	Vac6	Sc1	P24	Vac6	Sc4	P42	Vac8	Sc6
P7	Vac0	Sc2	P25	Vac0	Sc5	P43	Vac9	Sc6
P8	Vac2	Sc2	P26	Vac2	Sc5	P44	Vac10	Sc6
P9	Vac3	Sc2	P27	Vac3	Sc5	P45	Vac0	Sc7
P10	Vac4	Sc2	P28	Vac4	Sc5	P46	Vac2	Sc7
P11	Vac5	Sc2	P29	Vac5	Sc5	P47	Vac3	Sc7
P12	Vac6	Sc2	P30	Vac6	Sc5	P48	Vac4	Sc7
P13	Vac1	Sc3	P31	Vac7	Sc5	P49	Vac5	Sc7
P14	Vac2	Sc3	P32	Vac8	Sc5	P50	Vac6	Sc7
P15	Vac3	Sc3	P33	Vac9	Sc5	P51	Vac7	Sc7
P16	Vac4	Sc3	P34	Vac10	Sc5	P52	Vac8	Sc7
P17	Vac5	Sc3	P35	Vac0	Sc6	P53	Vac9	Sc7
P18	Vac6	Sc3	P36	Vac2	Sc6	P54	Vac10	Sc7

To finish the characterization of the intervention layer, we specify the value of key model parameters. Sensitivity/specificity of cytology and HPV-DNA tests were obtained using information from the Colombian National Institute of Cancer [29]. Costs of vaccination and tests were estimated in Colombian Pesos (COP) [46], and the screening coverage was recovered from the National Demographic and Health Survey conducted by the Ministry of Health in 2015 [47]. Finally, the disability weights for CC are obtained from the Global Burden of Disease 2017 study [48, 49]. The values of all parameters are reported in Table 11 at the end of the document.

### Model validation

We validated the population predictions generated by our model by comparing them with the UN population projections [50].

**Fig. 6:**
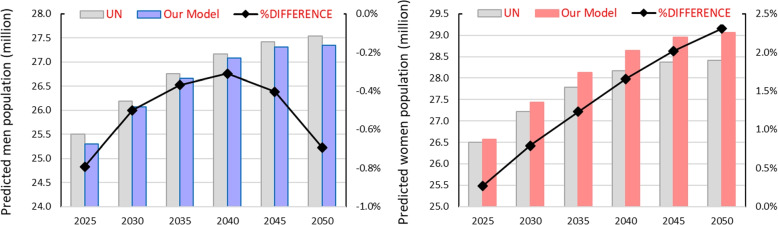
Predicted Colombian population and comparison with UN projections

Figure 6 displays the absolute values of the population for men (left) and for women (right), computed every five years along the 2020-2050 time horizon. It also shows the relative difference between the estimate provided by our model and the UN projections (plotted on the secondary vertical axis at the right). Only small differences can be appreciated in the totals (max deviation 2.3% at $t=$30years). Figure 6 also shows that the model tends to overestimate the size of the female population, and to underestimate the male one.

**Fig. 7:**
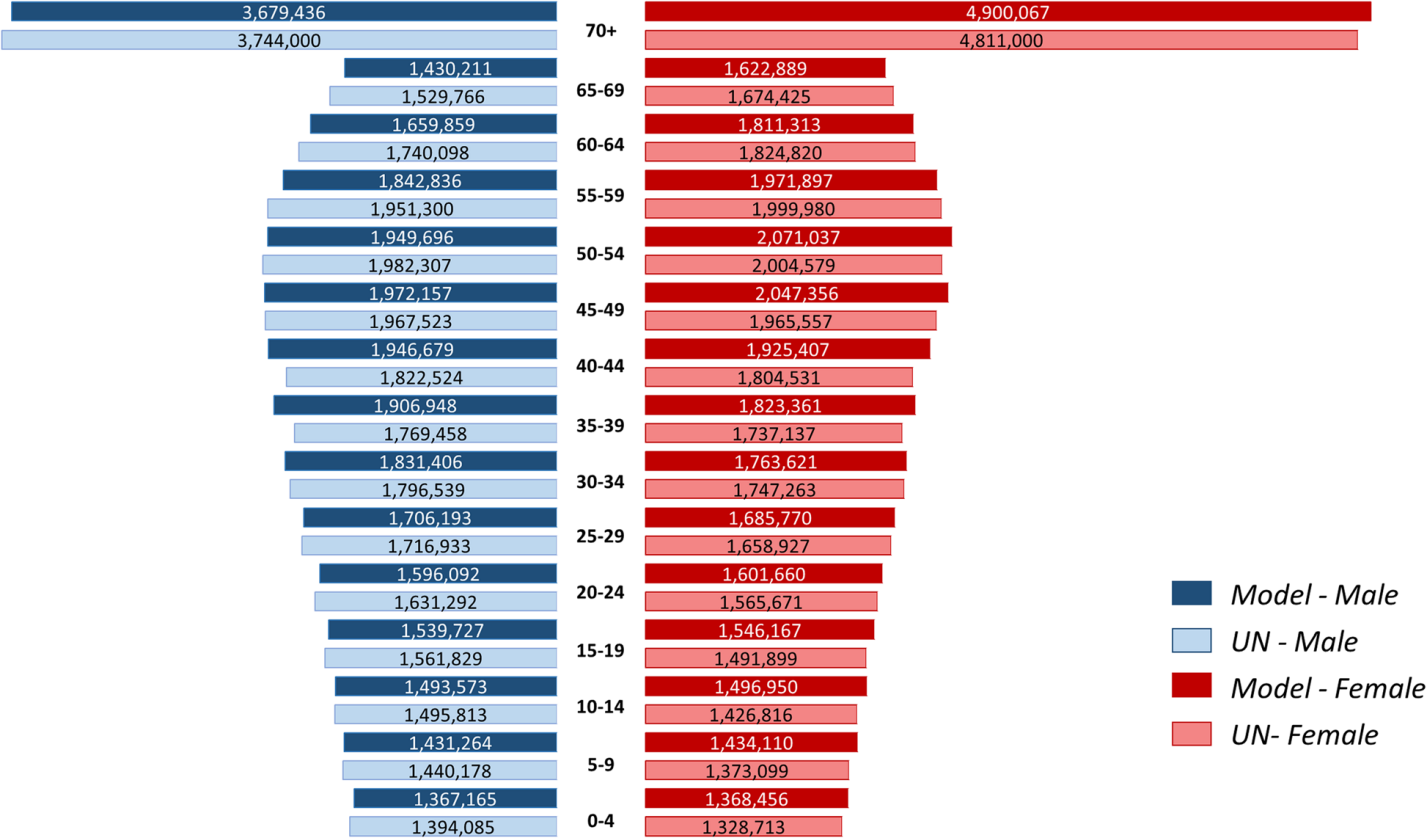
2050 Colombian population pyramid

To observe the discrepancies in more detail, in Fig. 7 we compare the Colombian male (left) and female (right) population pyramids predicted by our model for 2050 (darker hues) and the reference values (lighter hues). The most significant differences are for the population in the first and in the last age ranges, but are however within 5% in all cases. Thus, we can assume that our model is able to reproduce Colombian population dynamics.

As for the validation of the upper layers of the model, there are unfortunately very few projections available in the literature that can be used as reference. An available metric is the prediction of the Colombian age standardized mortality rate due to CC for 2019 [51]. Therefore, we estimated with our model the age standardized mortality rate for 2019 with a model whose $t=0$ is set to 2015, i.e. running the model along a five years time horizon, and compared it with the 2019 prediction in the literature. Our model provides an age standardized mortality rate of 6.628 for 2019, which falls within the 95% confidence interval (6.33–7.36) estimated in [51]. This increases our confidence about the ability of the model to properly represent the dynamics of CC in the Colombian population.

### Cost and effectiveness predictions

We report the predictions of cost and effectiveness for the 54 policies defined in Table 8, as obtained from the solution of the differential equations. We first review the *C*/*E* ratio, then we look into the disaggregated results by separately checking the two dimensions of cost and effectiveness. Finally, we conducted a sensitivity analysis to assess the robustness of policy ranking.

#### *C*/*E* scoring of policies

When comparing the *C*/*E* of policies P2-P54 with respect to the comparator policy P1, we first check whether some policies are definitely to be excluded from the analysis. In fact, there may be policies that cost more than P1 and do not reduce the DALYs. This is the case of 16 policies (P13-P18, P35, P37-P39, and P45-P50). An inspection of the details of the interventions defined for these policies reveals that they all have in common screening strategies that use the HPV-DNA test for both primary and secondary test, i.e. screening *Sc3*, *Sc6* and *Sc7*. Particularly, P13-P18 are policies implementing screening strategy *Sc3* (double HPV-DNA test, frequency scheme 3-5), and policies P45-P50 implement *Sc7* (double HPV-DNA test, frequency scheme 1-5). Policies P35, P37-P39 implement screening strategy *Sc6* (double HPV-DNA test, frequency scheme 3). Apart from P38, they all have in common a vaccination strategy that does not include women as a target (Vac0, Vac3, Vac5). These policies will not be further considered in this CEA.

**Fig. 8:**
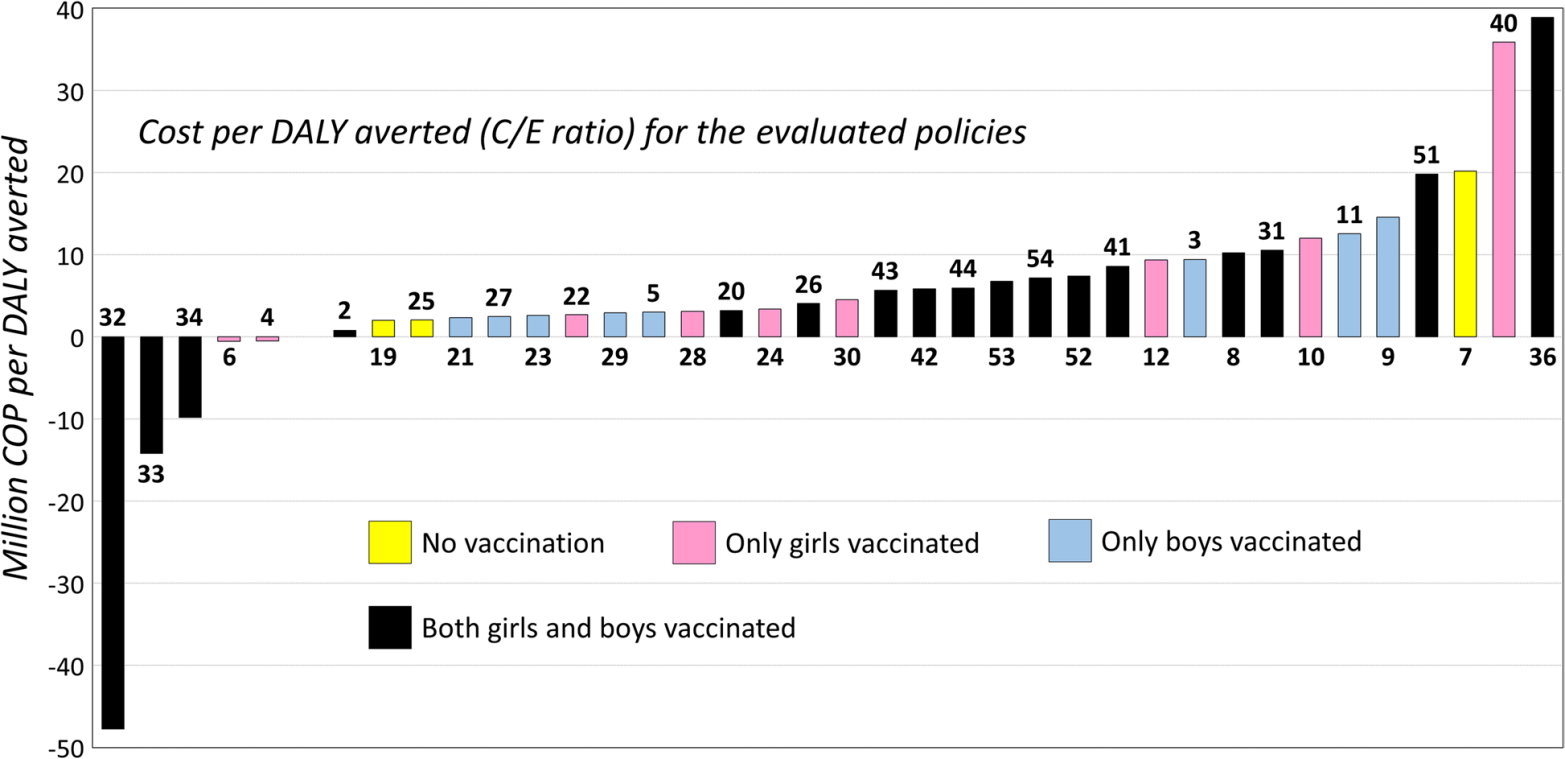
Cost-effectiveness ratio: cost (in million COP) per DALY averted

Figure 8 shows the cost-effectiveness ratio of all the remaining policies (those that are not certainly worse than P1, listed above), with respect to the comparator P1. Policies are sorted by their C/E, and a color scheme has been introduced to allow tracking the vaccination strategy they use.

As it can be observed from Fig. 8, there are five policies that have a negative C/E ratio. These policies achieve better results (less DALYs) at a lower cost. Therefore, replacing P1 with any of these five policies would imply a saving for a better effectiveness. It is interesting to notice that the best three policies (P32, P33 and P34) are all characterized by a vaccination strategy that targets both girls and boys. On the other hand, policy P36 at the rightmost extreme in Fig. 8 (highest C/E ratio) also considers vaccinating both sexes. Furthermore, there are policies with very low C/E ratio, such as P19 and P25, which do not include vaccination at all. These results show the importance of choosing the right combination of prevention and surveillance strategies when defining a public health policy for CC.

**Fig. 9:**
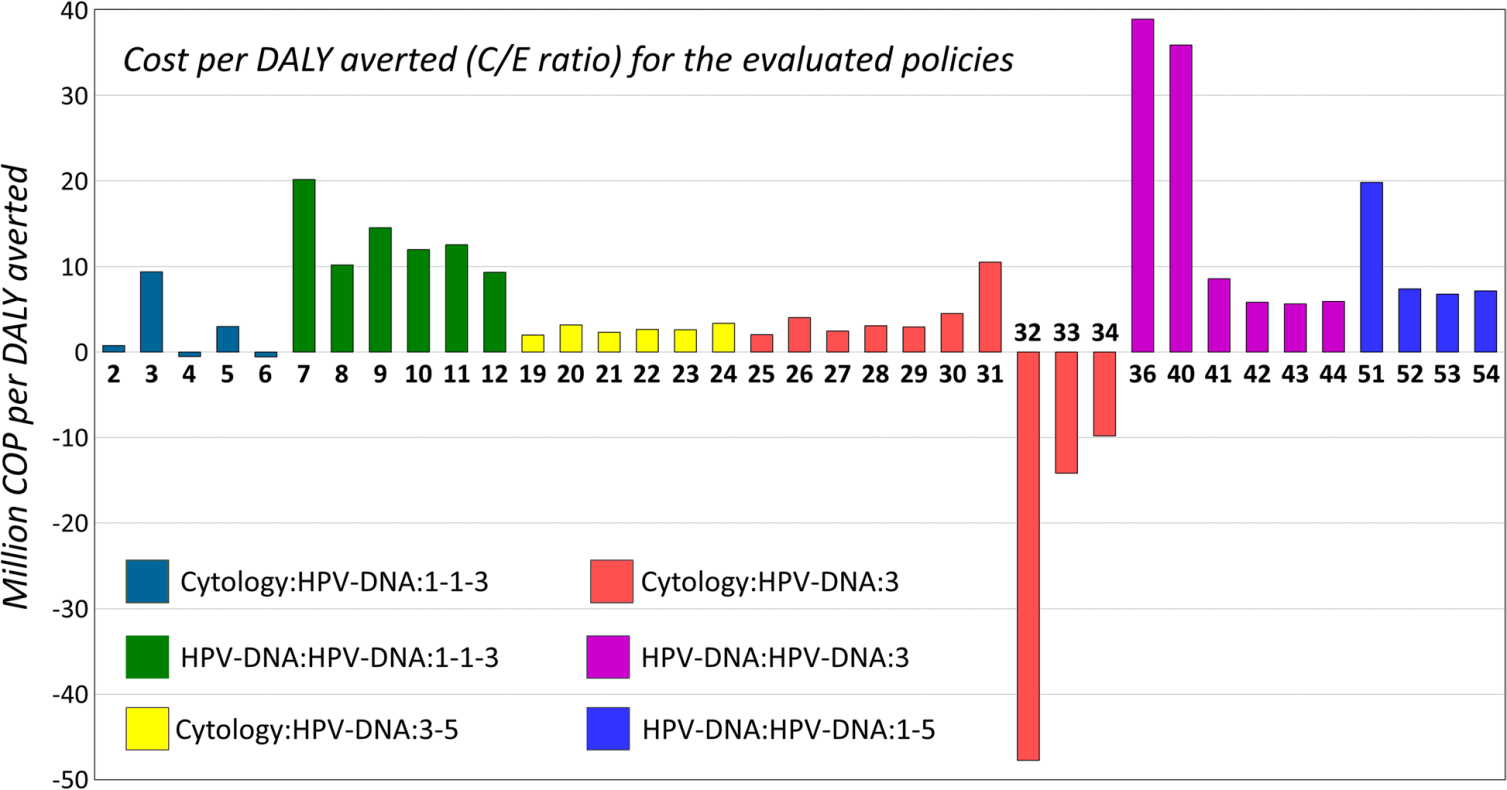
Cost-effectiveness ratio: cost (In million COP) per DALY averted

The bar chart in Fig. 9 reports the same data about the C/E of the policies, with a different sorting and color scheme, which now highlights the screening strategy. A fundamental conclusion obtained from Fig. 9 is that screening policies that use the HPV-DNA test for both primary and secondary test tend to be less cost effective than those that use cytology and HPV-DNA.

#### Disaggregated analysis

We separately analyze the cost and effectiveness of policies. Trading cost versus effectiveness allows taking into consideration different constraints and objectives of various stakeholders of the health systems; however, it introduces more complexity. The concept of *efficient frontier* can be applied to keep the analysis simple.

**Fig. 10:**
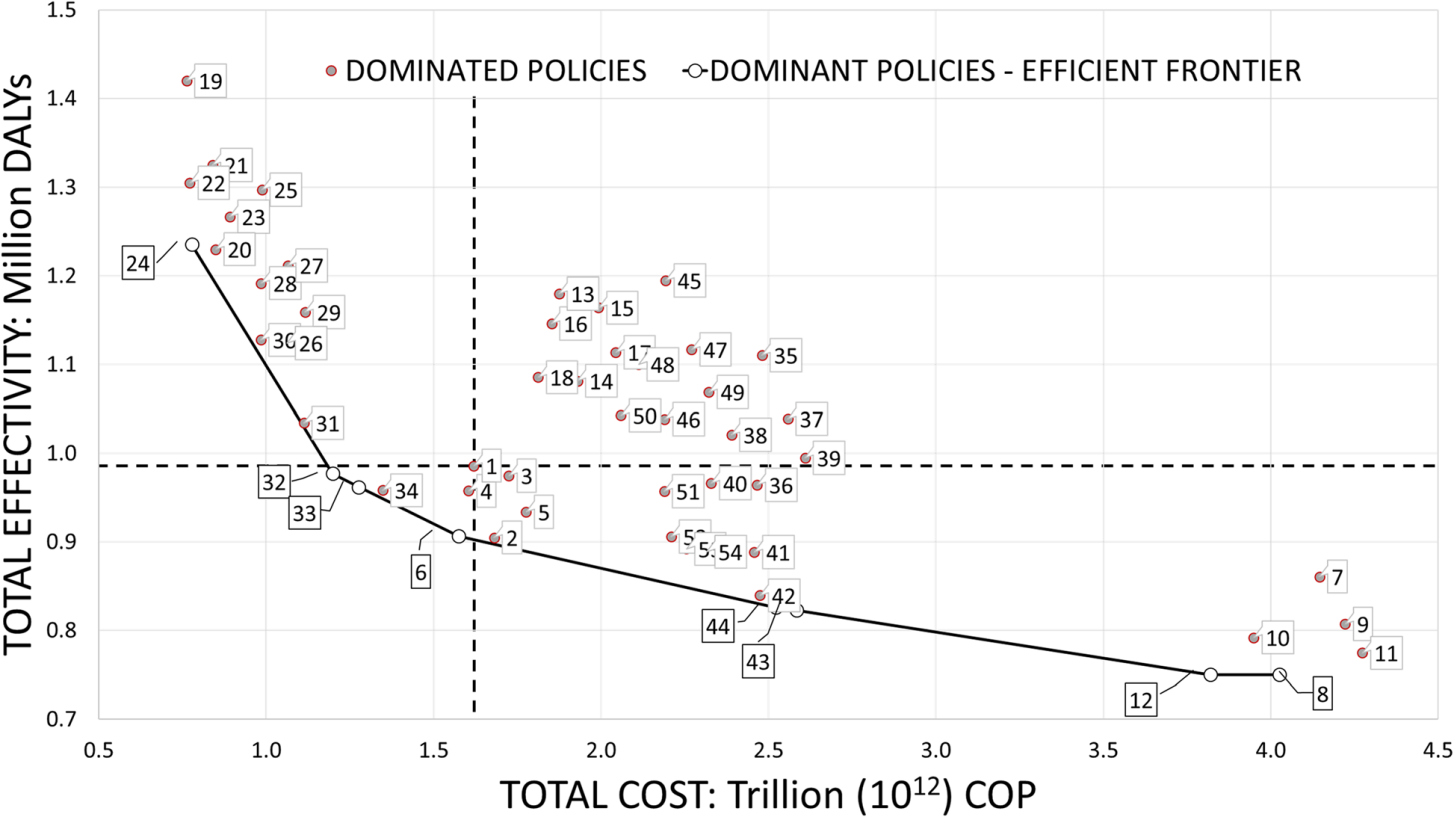
Disaggregated cost-effectiveness evaluation of policies: efficient frontier

Figure 10 plots results in the *cost-effectiveness plane*. Each policy is represented on the plane as a point, whose coordinates are given by its estimated cost (horizontal axis) and effectiveness (vertical axis). The ideal policy has low cost and high effectivity, i.e. reduced number of DALYs, which would be depicted as a point close to the origin.

The cost-effectiveness plane shows two additional dashed axes, centered at the comparator P1. Policies in quadrant I are those with higher cost and less effectivity than P1 (surely worse than P1), while those in quadrant III have less cost and higher effectivity (definitely better than P1). However, policies in quadrants II and IV may offer interesting trade-offs between expenditure and public health results.

Policies that are more clinically effective and cost-saving are called *dominant*, and identify the efficient frontier (the concave line in Fig. 10). The relative quality of a policy can be visualized on the cost-efficiency plane as its distance from the efficient frontier. Policies marked as red dots are said to be *dominated*, and have increased costs or reduced effectiveness compared to dominant strategies. Therefore, they will not be further considered in this analysis.

All the dominant strategies consider vaccinating solely women (24, 6, 12), or both women and men (32, 33, 43, 8). Policies that only vaccinate men or do not use vaccination are always dominated. Moreover, none of the policies that consider vaccination for the longest period of time (20 years) are within the efficient frontier. This happens because the benefits of vaccination campaigns are only observable after decades: vaccination intervention after year 15 of the analysis only adds to the costs without any significant returns in effectiveness.

Most of the dominant strategies consider screening with Pap test as a primary test (24, 32, 33, 6). Additionally, the most expensive and effective policies (12 and 8) are the ones with the highest screening frequency. The least expensive and least effective policies are those with the lower frequency between screenings (24 and 32).

Table 9 shows the ICER analysis for all the dominant strategies. Costs are in billion COP (Colombian Peso), over 30 years discounted at 3%. Health outcomes are in thousand DALYs, over 30 years.

**Table 9:** Analysis of dominant policies: costs over 30 years, discounted at 3% per year in billion COPs; health ouctomes over 30 years, in thousand DALYs

Policy	Cost (COPs)	Health outcomes (DALYs)	ICER (COPs/DALY)
24	777.897	1,236.512	0.630
32	1,198.650	977.216	1.629
33	1,275.958	962.837	5.027
6	1,574.955	906.447	5.398
43	2,521.734	825.865	11.749
12	3,818.992	750.037	17.108
8	4,025.723	750.006	6,676.876

Policy 24 has the lowest cost and the highest DALYs, and its ICER of 0.630 tells us that for averting an additional thousand DALYs, an investment of 0.630 billion COPs is required. In contrast, policy 12 (higher cost and lower DALYs) has an ICER of 17.108 implying that to avert one additional thousand DALYs, a much higher investment is necessary. Furthermore, when the investment is over 3,818.992 billion COP, an additional investment only marginally improves the effectiveness (policy 8).

**Fig. 11:**
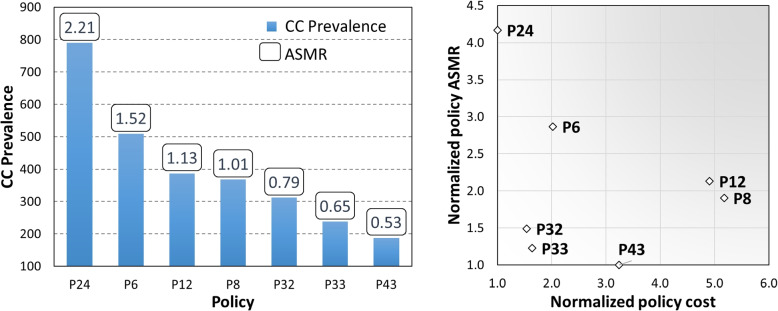
Predictions at year 2050: CC prevalence and ASMR for the dominant policies

We also report two epidemiologic health outcomes that can provide a different perspective on the effectiveness of policies: the prevalence of CC and the age-standardized mortality rate (ASMR)^1^. Both metrics are evaluated at year 2050. As it can be appreciated in Fig. 11, left chart, the ASMR is directly proportional to CC prevalence in the population. Among the dominant policies, those that only vaccinate women (24, 6, 12) have a higher prevalence and ASMR than those that also consider vaccinating males. The right chart in Fig. 11 shows the relationship between the predicted policy cost (horizontal axis) and the ASMR (vertical axis) for the dominant policies. Each variable is normalized with respect to the value of the best policy, i.e. the one achieving the minimal cost (P24) and the minimal ASMR (P43). Policies that are at minimal distance from the bottom-left corner of the chart have both low ASMR and costs that are comparable with the least expensive ones. For instance, the cost of policy P32 (P33) is only 1.5 (1.7) times the one of the least expensive one (P24), but it provides 3 (4) times better ASMR.

Finally, we review the results of the 1-1-3 CC screening policy that was used in Colombia until recent years (comparator policy P1). Table 10 shows the estimated prevalence, number of deaths, age-standardized mortality rate, and overall cost and effectiveness of this policy. As we observe, this policy is dominated and, aside from policy 24, all dominant policies have a lower estimated ASMR for 2050 than policy P1.

**Table 10:** Results for the 1-1-3 CC public health policy (P1). The estimated ASMR is per 100,000 women. The accumulated cost is presented in billions of COP, discounted at 3% per year. DALYs are discounted at 3% per year

	2020			2050			Overall time horizon	
Policy	Prevalence	Deaths	ASMR	Prevalence	Deaths	ASMR	Cost	Effectiveness
1	1,775	1,634	6.292	634	548	1.886	$1,619.356	986,025.73

#### Sensitivity analysis

The accuracy of the parameter estimation has to be always taken into consideration when making decisions based on the output of a predictive model. We assess the robustness of results by sensitivity analysis, checking whether and how the dominance relationships change with varying values of selected model parameters. The following parameters were considered for the sensitivity analysis:

1. Discount rate: commonly varied in CEA to evaluate the uncertainty associated with economic factors. It may be particularly relevant in the context of a developing country such as Colombia. Following various studies in the literature [11, 53, 54], we choose a range $[0-6]\%$.
2. Vaccination cost per dose: bound to the dynamics of big pharmaceutical companies and to the agreements that the government establishes with them. We choose a range of variation $[-10,10]\%$ with respect to the nominal value (reported in the Appendix).
3. Cost of testing: may change upon its widespread introduction. Currently, the cost of HPV-DNA test is higher than cytology. When the latest evaluation of the CC burden was conducted in Colombia, cytology was estimated to cost $8US, while a HPV-DNA test around $12US. We shall consider ranges of variation for both parameters, as defined in previous studies [30, 46].
4. Sensitivity of screening tests: highly correlated to processes, tools and expertise of health providers. A study conducted on a private health care facility in Bogotá [43] estimated the cytology sensitivity to lie in the interval [0.76-0.87] with 95% confidence level, a much higher value than the [0.3-0.85] interval reported by the Colombian National Cancer Institute [29]. For the purpose of our analysis, we choose the range [0.3-0.87] for cytology sensitivity.

**Fig. 12:**
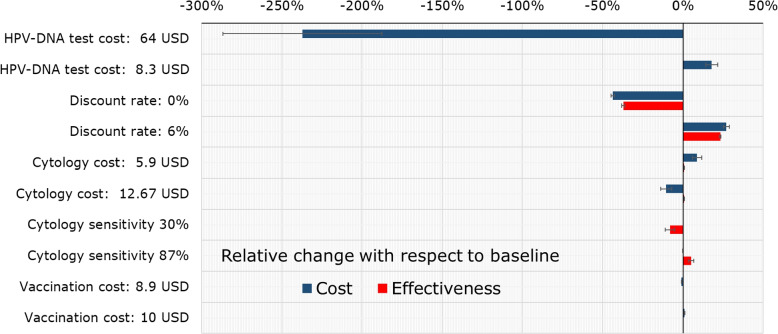
Average relative change of cost and effectiveness at the extremes of parameter variation ranges

The combined tornado diagram reported in Fig. 12 shows the variation (relative change with respect to the baseline) for cost (blue bars) and health outcome (red bars), averaged over the 54 policies. The error bars show the 95% confidence interval for the average variation across the considered policies. We can appreciate in Fig. 12 the importance of the discount rate and test cost variations. On the contrary, the considered changes in the cost of HPV vaccination appear to be not relevant for cost-effectiveness.

**Fig. 13:**
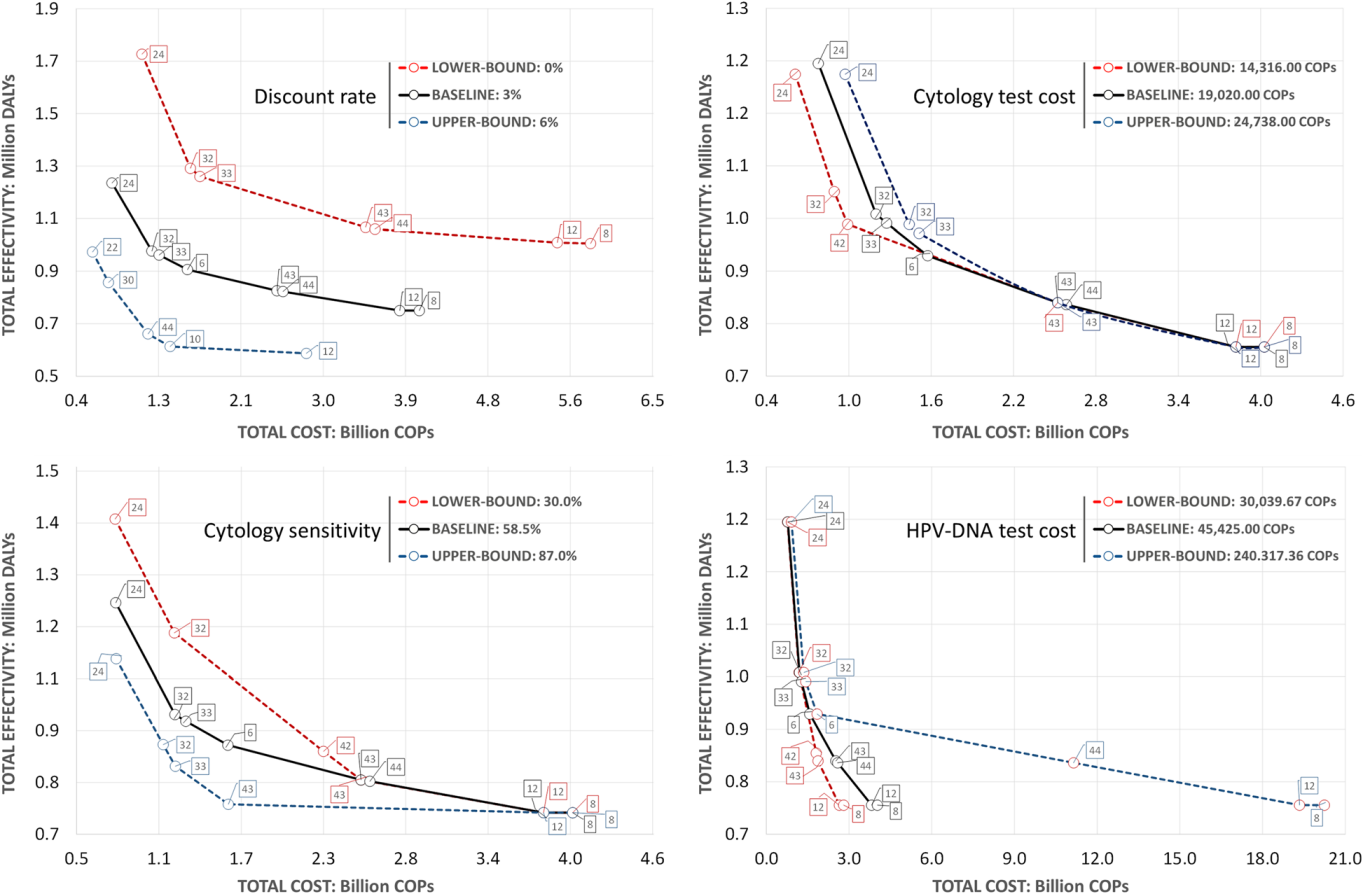
Variations of the efficient frontier when parameter values are changed

We show in Fig. 13 how the efficient frontier changes when the values of parameters are modified. The black lined frontier corresponds to the comparator scenario where all parameters take their nominal values (reported in the Appendix). The red frontier is calculated when the parameter is set to the smallest value within the sensitivity analysis range, while the blue one is the one estimated with the largest value in the range. We observe that policies 8, 12, 24 and 32 are robust in the sense that they belong in the efficient frontier in at least 10 of the 12 presented scenarios.

We observe that, although variations in the discount rate generate nominal changes in the total effectiveness, most of the efficient frontier policies remain the same. The same occurs with variations in the sensitivity of the Pap test. On the other hand, variations in the cost of the pap test do not appear to produce remarkable variations in the cost and effectiveness of the dominant policies. Indeed, most of the dominant policies continue being the same for the analyzed range. Finally, increasing the cost of the HPV-DNA test to the upper bound produces a significant increase in the total cost of policies 8, 12 and 44. This happens as the mentioned policies use the HPV-test for both primary and triage screening.

## Discussion

Screening and prevention policies against CC change over time and there is not consensus about an ideal program to be implemented around the world. In addition, the best intervention is not necessarily the one with the lowest *C*/*E* ratio, as countries usually have a limited budget to allocate to public health strategies. Moreover, there is not a single threshold for determining whether or not a *C*/*E* ratio is appropriate; therefore, healthcare decision-makers should conduct a detailed analysis that takes into account multiple factors while choosing the most appropriate strategies for intervention. A model-based approach can support flexible CEA of multiple policies for CC, and the automation of the analysis workflow into a software application relieves the burdens of data assimilation and model management, allowing decision-makers to focus on the analysis of costs and merits of candidate policies.

When tested on the Colombian case, our approach found that, in general, efficient policies include vaccination within the first 15 years and most of the dominant strategies consider screening with Pap test as the primary test. Screening alone tends to be insufficient, and it is recommended to have secondary prevention policies such as vaccination [54–56]. We found 8 dominant policies with costs ranging between $750,000 to $4,000,000 millions of COP and estimated efficiency (in DALYs) of 750,000 to 1,250,000 for the 30 years of simulation time. The obtained ICER for these policies was below $7,000 millions of COP per DALY averted. Moreover, for 2050 we estimated a reduction in the ASMR below 2.5 per 100.000 women if any of the policies on the efficient frontier is applied. In particular, policy P6, whose total cost is similar to that of the comparator policy P1 (has an ICER of $5.398 millions of COP per DALY averted and an estimated 1.521 ASMR for 2050). We performed sensitivity analysis to assess the impact of uncertainty in some key parameters, and found that policies 8, 12, 24 and 32 are robust in the sense that they belong to the efficient frontier in more than 80% of the evaluated scenarios.

To the best of our knowledge, this is the first attempt to evaluate integral policies that include screening and vaccination in Colombia. Previous studies analyzed the impact of screening or vaccination, but not the the combination of the two intervention types; and were unable to predict health outcomes in the long run [30, 51, 57]. With the support of our modeling approach, we can estimate the effects of different policies within a desired simulation time and evaluate them in terms of their cost and health outcomes. Our proposed methodology provides a framework to include male vaccination in addition to primary and secondary prevention policies, and a flexible usage by public health decision-makers, considering the automation of our modeling and analysis approach into an open software tool.

A notable advantage of our modeling methodology consists in encompassing a modular modeling approach with layers that can handle distinct concerns. As a consequence, the lowest layer model, i.e. the population model, can be reused and applied to the analysis of any disease. Nonetheless, the disease and intervention layers are specific to CC, as they encode the transmission and natural history of HPV infection, health states and intervention types that are only relevant for this condition.

There are several limitations to this study. First, we did not consider screening follow-up strategies, which consist in applying an additional test after a determined time-period for women who had a positive primary screening test but a negative triage test. These strategies have been adopted in some countries for preventing possible disease progression. Secondly, regarding vaccination, we did not consider the benefits of HPV vaccine against non-cervical conditions such as oral cancer, anogenital cancers, and genital warts. The inclusion of such effects would improve C/E ratios of interventions that consider vaccination. Moreover, we assume that when individuals who have not yet been exposed to HPV are vaccinated, they will acquire permanent immunity. However, the vaccine does not represent total protection, as it only protects for HPV types that are together responsible for up to 90% of cervical cancers [58]. Moreover, for approximations over longer time horizons, it is recommended to model female life expectancy by a time-dependent function. Equations for calculating the DALYs can be easily modified to include an approximation of a lifetime time-dependent function. Lastly, for countries where women enjoy an extended life expectancy, increasing the level of detail of the population compartmentalization for the upper age ranges would improve the accuracy of DALYs estimation.

## Conclusions

We propose a modeling methodology for the detailed estimation of costs and health outcomes of prevention and surveillance interventions for CC, at a country level. To do so, we build a continuous-time, deterministic representation of population dynamics, epidemics of disease, and interventions based on multiple layers of compartmentalized models. The population layer reproduces the natural processes of a population. The infection layer models the transmission and clearance of HPV infections within the population, as well as the progression and regression of the infection to precancerous lesions and eventually to CC. This layer describes the HPV transmission dynamics for both women and men, since both can acquire the infection and the incidence is proportional to the number of infected individuals in the population at any moment in time. Finally, the intervention layer models the effects of prevention and surveillance policies within the population.

We implemented our methodology into a software application built with R^®^ [12] and endowed with an interactive Shiny^©^ Web interface [13]. With this application, we analyzed a Colombian case study, evaluating 54 different policies based on the Colombian Ministry of Health guidelines for CC prevention, as well as CC policies that were considered in the CEA literature for low and middle-income countries. The results of our analyses identify a set of dominant policies that may be interesting candidates for decision-makers. We found that the screening and vaccination program deployed in Colombia until recent years is not included among the best policies.

Our method offers several practical advantages. It provides an automation of the modeling, parameter initialization and analysis into an open software tool, which facilitates the analysis of CC policies. Compared with traditional time-consuming discrete simulation models, our method can provide results for long time horizons in less time, for using a deterministic continuous approximation. Our approach provides flexibility for public health decision-makers to simultaneously explore multiple options of intervention and determine the policy to adopt among dominant strategies, according to cost constraints and desired health benefits. The implementation of the approach into an open software tool makes it attractive for a larger community of researchers and decision-makers.

## Model parameters and assigned values

Table 11 summarizes the values of parameters used in this paper for instantiating the models used for CEA.

**Table 11:** Model parameters

Parameter	Value	Source
Population initialization		[32]
Transition probability matrices		[30]
		[59]
Test accuracy estimates		
Cytology sensitivity	0.585	[29]
Cytology specificity	0.986	
HPV-DNA test sensitivity	0.925	
HPV-DNA test specificity	0.905	
Test and treatment costs		
Cytology cost	US$8.0	[46]
HPV-DNA test cost	US$12.10	[30]
Vaccination cost per dose	US$8.30	[30]
Adherence to screening schemes		
20-24 years	70%	[47]
25-29 years	78%	
30-34 years	83%	
35-44 years	85%	
45-49 years	86%	
Disability weights		
CIN1	0.049	[48, 49]
CIN2	0.049	
CIN3	0.049	
Cancer	0.451	

## Mathematical modeling details

In this section, we provide the full detail about the definition of the differential equations of the model.

### Population layer

By introducing the indicator function $I_X$ which takes a value of 1 when the predicate *X* is true and 0 otherwise, we can rewrite Eqs. 1, 2, and 3 of the population layer model in the following compact form:

11 $$\begin{aligned} \frac{dF_{a}(t)}{dt} =N(t)p_{f} I_{a=0}+\theta F_{a-1}(t) I_{a\ne 0}-\theta F_{a} (t) I_{a < K}-\mu ^{F}_{a} (t)F_{a}(t),\ a \in A \end{aligned}$$

12 $$\begin{aligned} \frac{dM_{a}(t)}{dt} =N(t)(1-p_{f}) I_{a=0}+\theta M_{a-1}(t)I_{a\ne 0}-\theta M_{a}(t)I_{a < K}-\mu ^{M}_{a} (t) M_{a}(t),\ a \in A \end{aligned}$$

Each equation includes a positive first term for births, which will only be non-zero for the first age range; a positive term for the individuals who came from an earlier age compartment, which is null for the first age range; a negative term for those who progress to the next age compartment (aging), which is null for the last age range *K*; and a negative term for the deaths. We do not consider incoming or outgoing immigration flows in this model. However, including them will not change the fundamental structure of the population model.

### Disease layer

Since the life expectancy depends on the health state of the individual, we enrich the characterization of death rates and we denote by $\mu _{a,h}^F(t)$ ($\mu _{a,h}^M(t)$) the death rate of women (men) in age range $a \in A$ and health state $h \in {HF}$ ($h \in {HM}$). We can now state in Eq. 13 the differential equations that model the dynamics of the disease layer for women, being those for men analogous in their structure.

13 $$\begin{aligned} \frac{dF^{h_1}_a(t)}{dt}= & {} N(t) p_f \mathbb{B}\mathbb{F}_{h_1}(t) I_{a=0} + \theta F^{h_1}_{a-1}(t)I_{a\ne 0}+ \sum \limits _{h_2 \in {HF}} F_a^{h_2}(t)\mathbb{P}\mathbb{F}_a^{h_2,h_1}(t) \nonumber \\- & {} \sum \limits _{h_2 \in {HF}} F_a^{h_1}(t)\mathbb{P}\mathbb{F}_a^{h_1,h_2}(t) -\theta F^{h_1}_a(t)I_{a\ne K} -\mu _{a,h_1}^F(t)F_a^{h_1}(t), \nonumber \\{} & {} a \in A, \ h_1 \in {HF} \end{aligned}$$

### Intervention layer

#### Vaccinations

For pulse vaccination interventions, we define the following parameters:

- [0, *T*] is the time horizon of model evolution, and $y : [0,T] \rightarrow \mathbb {N}$ is the function that returns the real year corresponding to time $t \in [0,T]$.
- ⓕ$_a^{y(t)}$ is the target percentage of women of age $a \in A$ to be vaccinated in year *y*(*t*) of the intervention.
- ⓜ$_a^{y(t)}$ is the target percentage of men of age $a \in A$ to be vaccinated in period *y*(*t*) of the intervention.

We update the set of health states for women to be $HF \cup \{V\}$ and the set for men to be $HM \cup \{V\}$.

We can now provide the differential equations that model the effects of vaccination interventions on the population. We first provide the equations that describe the dynamics of females on which the vaccination would be effective, i.e. whose health state belongs to ${HF}_V$:14The last term is accounting for the individuals who get vaccinated in year *y*(*t*) and change their health state as they become immune.

We can now generate the differential equations for the women who received the vaccination, i.e. whose health state is *V*.15Notice that the HPV immunity of individuals with health state *V* is modeled by making them not subject to the state change dynamics modeled by rate transition functions $\mathbb{P}\mathbb{F}_a$. No other compartments will be affected by vaccination interventions, as the vaccination would have no effect for women whose health state is not in ${HF}_V$. For the sake of brevity, we do not provide the corresponding equations for men, which are analogous to Eqs. 14 and 15.

#### Screening

Which health state women reach after an intervention depends on the treatment type. Women that undergo local treatment re-enter the health states transition dynamics followed by the general women population. We assume that, after treatment, women will reach a health state $h^* \not \in {HF}_V$, as they already acquired HPV infection. Women who undergo a hysterectomy will reach a new health state $\ddot{h}$, where they will no longer suffer from HPV infections and its consequences.

We can now provide the differential equations for modeling screening strategies. Differential equations for women of age $a \in A_S$ are presented below:

16 $$\begin{aligned}{} & {} \frac{dF^{h_1}{dt}_a(t)}{=} \theta F^{h_1}_{a-1}(t) + \sum \limits _{h_2 \in {HF}} F_a^{h_2}(t)\mathbb{P}\mathbb{F}_a^{h_2,h_1}(t) - \sum \limits _{h_2 \in {HF}} F_a^{h_1}(t)\mathbb{P}\mathbb{F}_a^{h_1,h_2}(t) \nonumber \\- & {} F^{h_1}_a(t)f_a d_a c^{+}_{i,j}I_{h_1 \in {HF}_L \cup {HF}_H} - \theta F^{h_1}_a(t)I_{a\ne K} -\mu _{a,h_1}^F(t)F^{h_1}_a(t), \quad a \in A_S \end{aligned}$$

Notice that we slightly simplified the general equation because we assume screening is not applicable to the first age range. The second negative term in Eq. 16 accounts for screening disease detection. Individuals who undergo screening (under a policy with frequency $f_a$ and adherence $d_a, \forall \ a \in A_S$) and are suffering from a condition that requires treatment ($h_1 \in {HF}_L \cup {HF}_H$), a correct diagnosis ($c^{+}_{i,j}$) will result in a health state change. The misdiagnosis of true positives ($1-c^{+}_{i,j}$) will not lead to a health state change and is therefore not explicitly represented. The incorrect diagnosis of true negatives is not relevant for health states transition dynamics, but will be considered when measuring policies costs.

We now provide the differential equations for women in state $h^* \not \in {HF}_V$:

17 $$\begin{aligned}{} & {} \frac{dF^{h^*}{dt}_a(t)}{dt}= \theta F^{h^*}_{a-1}(t) + \sum \limits _{h_1 \in {HF}} F_a^{h_1}(t)\mathbb{P}\mathbb{F}_a^{h_1,h^*}(t) + \sum \limits _{h_1 \in {HF}_L} F^{h_1}_a(t)f_a d_a c^{+}_{i,j} \nonumber \\- & {} \sum \limits _{h_1 \in {HF}} F_a^{h^*}(t)\mathbb{P}\mathbb{F}_a^{h^*,h_1}(t) - \theta F^{h^*}_a(t)I_{a\ne K} -\mu _{a,h^*}^F(t)F^{h^*}_a(t), \quad a \in A \end{aligned}$$

For state $$\ddot{h}$$:

18 $$\begin{aligned} \frac{dF^{\ddot{h}}_a(t)}{dt}= & {} \theta F^{\ddot{h}}_{a-1}(t) + \sum \limits _{h_1 \in {HF}_H} F^{h_1}_a(t)f_a d_a c^{+}_{i,j} -\theta F^{\ddot{h}}_a(t)I_{a\ne K} -\mu _{a,H}^F(t)F^{\ddot{h}}_a(t), \nonumber \\{} & {} a \in A \end{aligned}$$

## Data Availability

All data generated in this study are included in this published article. The complete input datasets, together with the source code of the software application that automates the modeling approach, and supports the generation and analyses of model outputs, is freely accessible on the Duke University Gitlab public repository located at https://gitlab.oit.duke.edu/im90/cervicalcancer. A README file that provides a complete user guide can also be found in the above mentioned repository.

## Abbreviations

ASMR: Age-standardized mortality rate
CC: Cervival Cancer
CEA: Cost-effectiveness analysis
C/E ratio: Cost-effectiveness ratio
CIN: Cervical Intraepithelial Neoplasia
COP: Colombian Peso
DALY: Disability-Adjusted Life Years
DANE: Colombian National Department of Statistics
HPV: Human Papillomavirus
ICER: Incremental cost-effectiveness ratio
QALY: Quality-Adjusted Life Years
YLD: Years Lived with Disability
YLL: Years Lost Life
UN: United Nations
WHO: World Health Organization

## Footnotes

1 The ASMR is the weighted average of the age-specific mortality rates, where the weights are the proportions of persons in each age group [52]
